# The effectiveness of a behavioral science and design intervention for family savings on use of maternal health services and male involvement: study protocol for a randomized controlled trial

**DOI:** 10.1186/s12889-022-13985-1

**Published:** 2022-08-19

**Authors:** Lisa Hartwig, Victoria Namukose, Junko Kiriya, Chrispinus Onyancha, Akira Shibanuma, Masamine Jimba

**Affiliations:** 1 Department of Community and Global Health, School of International Health, Graduate School of Medicine, The University of Tokyo, 7-3-1 Hongo, Bunkyo-ku, Tokyo, 113-0033 Japan; 2 clinicPesa Limited, P.O. Box 26512, Kampala, Uganda

**Keywords:** Maternal health, Male involvement, Savings, Mobile money, Behavioral science

## Abstract

**Background:**

Lack of financial preparedness for pregnancy can lead to adverse outcomes during childbirth. Behavioral science interventions have been shown to influence savings behavior. Financial savings interventions can be adapted for the purpose of encouraging individuals to save towards maternal healthcare costs. This article describes a protocol to assess the effectiveness of an intervention formulated with a behavioral science approach for encouraging use of maternal health services through increased financial savings for birth preparedness and maternal healthcare costs among pregnant women or their partners in Uganda.

**Methods:**

A randomized controlled trial will be conducted to assess the effectiveness of the intervention among pregnant women or their partners in Uganda’s central region, including the capital of Kampala. Seven hundred pregnant women (12–35 gestational weeks) or their partners will be recruited. All participants will receive access to a committed mobile money health savings account provided by a local organization that also offers savings targets and reminders for antenatal care appointments and health tips as part of a “Mamas Program” offered to expectant mothers. The time period in the intervention is from the day of enrollment until two weeks after the delivery date. The control group will receive the standard Mama Program offering. The intervention group will receive the standard Mama Program offering plus behavioral designs encouraging savings behavior through short-message service (SMS) text messages. The primary outcome is usage of maternal health services measured by level of birth preparedness and delivery at a health facility. Secondary outcomes include male involvement in maternal healthcare, measured by financial support, as well as total savings for healthcare, assessed using the validated amount of savings accrued in participants’ clinicPesa accounts from the day of enrollment plus any withdrawals for healthcare expenditures during the intervention period.

**Discussion:**

The study will contribute to a better understanding of the effectiveness of behavioral designs encouraging financial savings during pregnancy into committed mobile money health savings accounts. The study could contribute to demonstrating the effectiveness of savings on birth preparedness, usage of maternal health services, and male involvement in maternal healthcare.

**Trial registration:**

UMIN-CTR Clinical Trial, UMIN000046472. Registered on 19 January 2022. https://center6.umin.ac.jp/cgi-open-bin/icdr_e/ctr_view.cgi?recptno=R000053008

**Supplementary Information:**

The online version contains supplementary material available at 10.1186/s12889-022-13985-1.

## Background

Maternal mortality is a common problem worldwide despite high levels of investment and effort over the last several decades, especially in low and middle-income countries [1]. The first target under Sustainable Development Goal 3, “Good Health and Well-being,” is to reduce the global maternal mortality ratio (MMR) to less than 70 per 100,000 births, indicating its importance for development and progress towards attaining universal health coverage and human rights through health [2]. MMR is 211 per 100,000 live births worldwide, and sub-Saharan Africa (SSA) carries two-thirds of the global burden of MMR [1].

Thaddeus and Maine developed the “Three Delays Model,” which describes the three main factors that affect a mother’s decision to seek treatment for delivery: 1) delaying to seek care, 2) delaying arrival at a health facility, and 3) delaying the provision of adequate care [3]. Educational attainment, a well-functioning health system, and economic factors are all associated with maternal mortality particularly in SSA [4]. In particular, financial and opportunity costs are often cited as a demand side access barriers for women and families [5, 6].

There have been many demand side and supply side interventions to promote increased access and uptake of maternal health services [3, 4]. Interventions that increase antenatal care (ANC) visits before childbirth and skilled birth attendance (SBA) are widely known to improve maternal health outcomes [7, 8]. However, more use does not necessarily mean better outcomes due to quality of care provided at the facility [9]. Frequently, women in low-resource settings opt to deliver at home due to perceived cost or quality of care at a facility [10]. By improving the financial preparedness of a mother and family, the financial barrier for access to maternal health services and birth preparedness can be overcome.

Lack of financial preparedness for pregnancy can lead to adverse outcomes during childbirth [1]. Because a mother and her partner have not accounted for the costs of the pregnancy, they are less prepared if they encounter an emergency during childbirth. Promoting antenatal care visits or providing vouchers for transportation have been shown to increase skilled birth attendance and delivery in a hospital setting, but the effects are reduced once financial support is removed and is unsustainable [11]. Thus, more emphasis must be placed on increasing financial empowerment when it comes to spending money on healthcare.

Male heads of household are often the primary decision maker when it comes to family decisions in SSA, particularly decisions surrounding finances or healthcare [12]. Many studies have reported positive associations between male involvement in maternal healthcare and maternal health outcomes, such as male partner attendance at ANC, increased utilization of SBA, and reduction in post-partum depression [12–16]. Male involvement can also include financial support for transport or appointments in SSA [17]. Yet male involvement is still not commonplace, and financial barriers are often cited as a reason for not seeking maternal healthcare [5, 6]. Interventions that cluster other health services such as HIV testing with ANC or training healthcare workers to be more male-inclusive have shown positive effects in involving the male partner [18–20]. Most interventions are focused on education, such as teaching the husband about danger signs during a pregnancy so that they may seek early care or the timing of ANC appointments [15, 21, 22]. There is an opportunity to create a behaviorally informed intervention that encourages increased male involvement in maternal healthcare.

Behavioral science has had increasing influences in the field of public policy and public health, especially in terms of savings behavior. For example, behavioral “nudges” and “choice architecture” are cited as encouraging the public to change their behavior without extreme regulation, such as setting defaults on retirement savings accounts at workplaces [23]. Behavioral “insights” are defined as “an inductive approach to policy making that combines insights from psychology, cognitive science, and social science with empirically-tested results to discover how humans actually make choices” [24]. In SSA, behavioral approaches have been tested for motivating savings behavior and results have shown that physical reminders, such as a coin-shaped object where the participant tracks their own savings progress, have the most impact on savings behavior; text messages that sound like they are from their child had a marked improvement on savings behavior as well [25].

Financial savings interventions have also been used to improve health outcomes, although they are typically unsustainable in nature due to provision of vouchers or conditional cash transfer (CCT) programs [26]. For example, a study in India determined that a CCT program for increasing births in health facilities had a significant impact in the community [27]. A systematic review for CCTs to improve uptake of health interventions determined that although effective, the success of the CCT depends on the efficacy of the local service provision and infrastructure [28]. There is compelling evidence that well-designed financial interventions can improve health outcomes, but a reliance on external funds or external systems can reduce the impact.

Financial savings interventions can be adapted for encouraging individuals to save towards maternal health care costs. Approximately 12% of the world’s population spends at least 10% of their household budgets to pay for health care [29]. Even with increased public spending, service coverage of maternal, newborn, reproductive, and child healthcare is still low in low-income countries, and out of pocket expenditure is still high, especially in SSA [29]. Financial literacy overall is low in sub-Saharan Africa, and gender and power dynamics around spending decisions impact savings behavior [30]. Financial literacy interventions have limited impact and effect especially over the long term based on a systematic review, and recommendations are made to design savings devices with choice architecture and behavioral insights in mind [31].

Mobile money accounts are widely used in lieu of traditional banking in SSA [32]. Savings promotion trials generally increase savings rates in the region [33]. Training on financial literacy is not enough to encourage savings for maternal health care costs as the effectiveness of trainings on financial literacy is negligible [31]. Furthermore, individuals may still spend their savings on household expenses or other goods rather than maternal health care costs. There is limited evidence of savings interventions focused on health care [33].

In Uganda, the maternal mortality rate (MMR) stands at 336 per 100,000 live births [34]. The government contributes approximately 16% of the total cost of health expenditure with no social health insurance programs. Thus, Ugandan citizens typically pay a high out of pocket expenditure, which is associated with high financial risk. Furthermore, less than 0.7% of Uganda’s 42 million have adequate healthcare insurance coverage [34]. When considering the challenges in covering costs of pregnancy and delivery, there is an opportunity for couples to start saving earlier in preparation for a highly anticipated event. Small incremental amounts can add up and become future options, such as when couples would prefer to have a choice in selecting where to deliver their child to receive higher quality services [11]. Expectant mothers also might be expected to contribute up to 100,000 Ugandan Shillings (approximately USD 30) when delivering at a facility, which most mothers cannot afford due to the subsistence nature of their income earnings [11].

Male involvement in maternal healthcare in Uganda is in alignment with the findings about the SSA region in general, namely that positive outcomes are associated with increased male involvement but men are still not heavily involved [35]. Factors associated with increased attendance to ANC in one region were receiving information from a healthcare worker, knowledge of ANC services, and SBA at last delivery [36]. Interventions were focused on encouraging male attendance with HIV testing appointments or encouraging male-inclusive family planning services [20, 37]. Yet opinion leaders from key informant interviews suggested that without external financial aid, these interventions are not sustainable [38]. When men control the household finances and make decisions of households, finding a way to empower them to be involved on a voluntary and willing basis can lead to improved outcomes for maternal healthcare.

Out of 26 million mobile subscribers in 2020, approximately 7.4 million are using smartphones with the numbers increasing year over year [39]. Furthermore, mobile money is widely used in SSA with more than 9 million users registered in Uganda alone [40]. Thus, mobile money accounts are a suitable mechanism for encouraging savings in contexts where bank accessibility might be low to include the most vulnerable and hard-to-reach populations. Financial savings interventions in Uganda showed that incentives could increase the number of deposits and diversification of assets by families [41]. The problem is that pregnant women and families in Uganda are not financially prepared and empowered to access quality and timely maternal health care.

It is possible to see how savings interventions can be applied to the field of healthcare, and technology can also expand upon the possibilities for empowering people in Uganda. This study intends to apply behavioral designs through mobile money technology and short message service (SMS) text messages to encourage couples to save early in their pregnancy for birth preparation and potential emergencies. A local company registered in Uganda runs committed mobile money health savings accounts and will partner on recruiting participants and collecting data for the research.

### Objectives

The primary objective of this study is to assess the effectiveness of a behavioral intervention designed to encourage birth preparedness through financial savings for healthcare costs among pregnant women and their partners in Uganda. The study will also examine whether increased earmarked financial savings for healthcare costs lead to increased utilization of maternal health services and male involvement in maternal healthcare.

## Methods

### Study setting

The study will be conducted in Kampala and Wakiso districts in the central region of Uganda, a low-income country [42]. The study area was selected due to the high population density and varied housing patterns with urban, semi-urban, and rural areas. The population of the region is 3,504,498 (Kampala: 1,507,080; Wakiso: 1,997,418) [34]. The region has 2047 health facilities (Kampala: 1458; Wakiso 589) and 74.3% coverage of mobile phone ownership [34].

### Study design

**Fig. 1:**
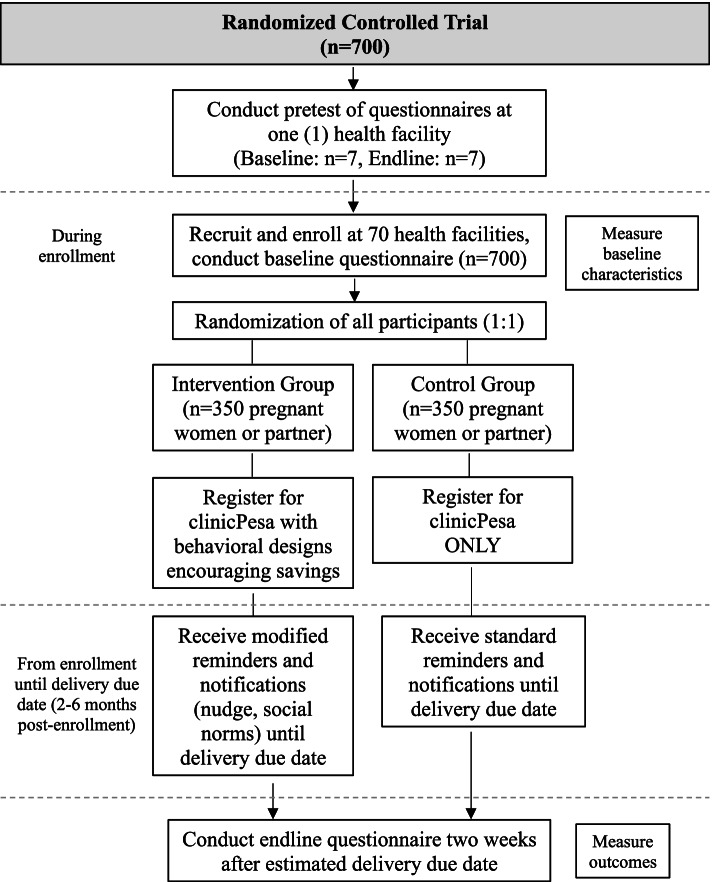
Study flow chart

**Fig. 2:**
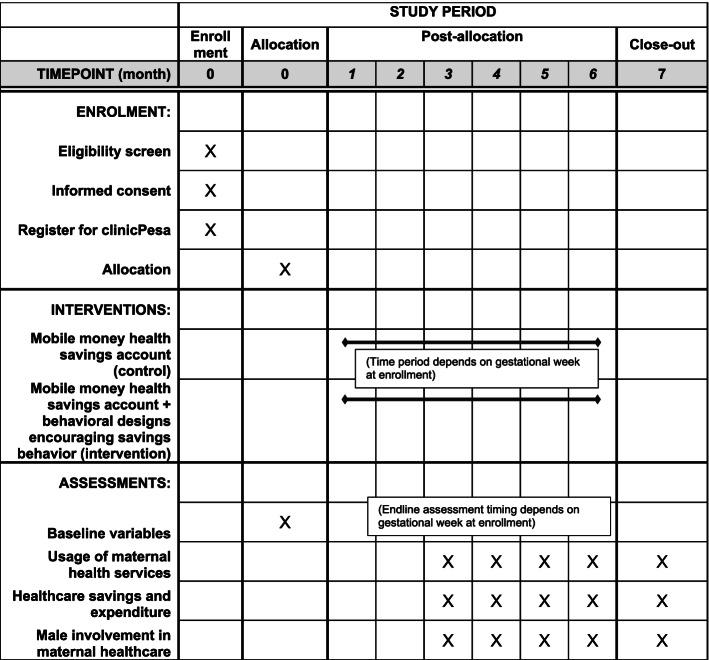
SPIRIT figure

We will conduct a randomized controlled trial (RCT) to test the effectiveness of the intervention comprised of a behaviorally informed mobile money health savings account on birth preparedness, maternal health service utilization, male involvement in maternal health, and savings amount towards healthcare costs. A dedicated mobile money health savings account will be provided by clinicPesa, a mobile money health savings company registered in Uganda. clinicPesa offers a commitment device for savings, where a client’s savings can only be spent on healthcare expenditures and not withdrawn for other purposes. The overall study flow chart is shown in Fig. 1. The schedule of the RCT is shown in the Standard Protocol Items: Recommendations for Intervention Trials (SPIRIT) figure (Fig. 2). The SPIRIT checklist is provided in Additional file 1.

### Sample size, eligibility, and recruitment

The minimum sample size for the RCT is 572 participants (286 in each arm). It was calculated using OpenEpi version 3. The power of the study was set at 80% and the significance level at 5%. A previous savings group research in a SSA region for maternal healthcare service utilization was used for assumptions [43]. Considering an approximate 20% dropout rate and sampling unit rounding, 700 participants will be recruited (350 participants in each arm).

To be eligible for joining in this study, participants should have the following characteristics: (1) 18–49 years old, (2) between 12–35 gestational weeks (pregnant female) OR partner of someone who is (male), (3) own a mobile phone, (4) has a registered Mobile Telephone Network (MTN) mobile money account, (5) willingness to enroll in clinicPesa’s health savings mobile money account. Participants are ineligible if their partner is known to already be participating in the study to avoid duplicating results of savings amounts; only one member per household may participate in the study.

Participants will be recruited and enrolled in person by clinicPesa at 77 public and private health facilities where clinicPesa operates. Five trained research assistants will approach male and female clients at the maternity ward of selected health facilities. The clients at the health facility will be offered an opportunity to enroll in the study when they sign up to join clinicPesa. In the case of a COVID-19 lockdown on movement, participants will be recruited through a phone query from clinicPesa’s registered user base. Users who sign up for clinicPesa agree to terms and conditions set by clinicPesa, which include the possibility of being offered to join studies to improve the product offered by clinicPesa.

### Randomization

A person who is not involved with any other study procedure (including recruitment, implementation of the intervention, and data analysis) will share a computer-generated randomized allocation table with clinicPesa at the beginning of the recruitment period, which will not be shared with the study recruitment team [44]. At the end of each recruiting day, the customer ID numbers of participants who agreed to join the study will be matched to the table to be assigned to either the control or the intervention group (1:1 randomization), and a study participant ID will be created to maintain participant anonymity within the study [32].

### Blinding

Participants will receive a description of the study during the informed consent process, but they will not be told which group they have been allocated to after enrollment. The study recruiters at the beginning of data collection and outcome assessors during final data collection will be blinded to the allocation. In addition, the data analyst will be blinded to the allocation of the participants until after the data analysis is complete.

### Intervention

The intervention consists of a basic mobile money health savings account as part of a Mamas Program offered by the local partner organization administering mobile money health savings accounts and additional behavioral designs to encourage savings for maternal health costs. The intervention was formulated using behavioral science approaches for promoting health savings towards the costs of maternal health care among pregnant women and their partners. The Mamas Program is a subset of the company’s clients that are registered as pregnant, and they receive additional reminders on ANC appointments, nutritional tips, and health tips about pregnancy. In the intervention group, participants receive the standard Mamas Program local company offering and messaging in addition to behavioral designs that encourage saving. The behavioral designs include “nudges” to encourage saving behavior; this will be done through reminder messages that sound like they are from their child, notifications about meeting their targets, and social norms notifications based on their savings rates and time in the intervention.

In the control group, participants receive the standard Mamas Program local company offering and messaging only. Both groups will have access to a mobile money health savings account as offered local company with the ability to deposit and withdraw health savings or set targets related to pregnancy for their savings goals, in addition to the Mamas Program messages for a healthy pregnancy.

It is assumed that the risk of contamination is low because participants are unlikely to share financial or account information with others, including any messages received from clinicPesa to an individual phone number. Participants can voluntarily withdraw from the study at any time.

### Outcomes

The primary outcome is level of birth preparedness and usage of maternal health services, assessed by the number of birth kits or items within birth kits purchased, antenatal care visits, skilled birth attendance by a doctor, nurse, midwife, and/or medical assistant or clinical officer, and delivery at a health facility, all based on recall by the participant; the amount of validated expenditure in participants’ clinicPesa accounts from the day of enrollment during the intervention period will also be assessed. The primary outcome will be measured by asking the participant during the end-line survey about their pregnancy and delivery experience.

Secondary outcomes include male involvement in maternal healthcare, measured by financial support, attendance to ANC, assisting with planning the delivery, and providing perceived emotional support to the mother, as well as total savings for healthcare, assessed using the validated amount of savings accrued in participants’ clinicPesa accounts from the day of enrollment plus any withdrawals for healthcare expenditures during the intervention period. The withdrawals will also be coded to determine if they were used for maternal health service costs, including transport, or other healthcare costs, such as birth preparedness kits, baby materials, or others. Deposits into the account will be coded by whether it was the mobile money account owner or another mobile money account (for example, a different family member saving into the account).

Male involvement in maternal healthcare will be assessed by measuring whether they provided financial support for clinicPesa or otherwise, attendance to ANC, and assisting with planning of the delivery, measured by an ad-hoc male involvement index [45]. Women will be queried about the perceived levels of involvement, and men will be queried about their level of involvement.

### Other information

The following information will be collected during the baseline interviews to conduct sub-group analyses and to identify factors associated with savings for maternal healthcare and male involvement during pregnancy: age, education, employment, area of residence (rural or urban), pregnancy intention, joint decision making within a couple. Other information, such as feedback on the intervention, will be collected to inform future interventions, but this information will not be part of the analysis for this study.

### Data collection

On the day of enrollment after consenting to join the study, all participants will fill out an interviewer-administered survey conducted by research assistants to identify baseline characteristics, demographic information, savings intention and behavior, and knowledge of and involvement in maternal health. This information will be used to conduct descriptive statistics and sub-group analyses to identify factors associated with savings behavior: age, education, marital status, employment, rural/ urban status, estimated wealth quintile, pregnancy status, attitudes towards male involvement in pregnancy, attitudes of and behaviors towards saving money, pregnancy preparation intentions.

Once a woman delivers, data on the amount saved plus any expenditures during the period of the intervention will be pulled from clinicPesa’s database for registered participants [46, 47]. The participants will also be called for an end-line questionnaire two weeks after their estimated delivery date that collects the same information as baseline with additional items on how they used the clinicPesa platform, delivery experience, and perceived involvement of the male partner in maternal healthcare. A pre-test of the data collection tools will be conducted with 14 random participants users from clinicPesa’s user base.

### Data analysis

Background characteristics will be analyzed to determine that both the control and intervention groups were balanced and randomization was done correctly. The primary outcome of birth preparedness and maternal health utilization will be compared between the intervention and control groups. The mean total savings amounts of participants (regardless of expenditure) will be compared between the intervention and the control groups using T-tests for mean savings. Mixed-effect regression analysis will be performed to assess the effect of the intervention on the following outcomes: amount of money saved across the study period (plus withdrawals), birth preparedness, maternal health service utilization, male involvement in maternal healthcare, and utilization of money for pregnancy-related health costs (delivery, preparation, transportation, or other, if applicable). Logistic regression will be used to control for potential cluster effects. Weighting approach will be used to control for variation in probability of participants being included in the study across study recruitment sites (large facilities vs small facilities).

It is expected that after a participant enrolls in the study and is randomized, final savings data can be collected remotely even if the participant does not engage with the platform (i.e., does not save any money after enrollment) or complete the end-line survey. In this case, savings outcome data can still be collected. Attrition rates and reasons for dropout will be logged if possible. An intention to treat (ITT) analysis will be conducted, and a sensitivity analysis will be conducted for any missing data [48, 49]. The significance level will be set at 5%, and Stata version 13.1 will be used for analysis of the data set.

## Discussion

The research study may demonstrate that behavioral insights can be used to financially empower mothers or their partners in Uganda to save towards pregnancy, thereby improving birth preparedness and other health outcomes. This approach could be an ideal option when encouraging future parents to save for their family as it is embedded into an existing infrastructure of the country.

There are several limitations in the present study. First, the local partner organization at time of publication is only registered to operate with private health facilities in the central region of Uganda, which is highly populous and excludes those who live in rural areas outside of Kampala. Considering the prevalence of maternal mortality in other regions of Uganda and persons attending public health facilities for health care, the study likely does not include hard-to-reach populations. Second, the mobile money health savings account validated amounts does not include savings that could be saved elsewhere, such as in cash or a traditional bank account. While the end-line questionnaire attempts to account for this, amounts could be inaccurate due to recall bias and social desirability with savings amounts outside of the mobile money savings account stated as higher than they are. Third, some participants may be lost to follow up because the end-line questionnaire is phone-based. However, a phone-based end-line survey was determined to be suitable for post-delivery, and the COVID-19 pandemic may also restrict in-person meetings. Lastly, findings of the study are focused on one locality and could be transferrable to other countries or organizations only if they also operate a committed mobile money health savings account.

The results would provide the Government of Uganda and other development organizations with evidence for strengthening programs focused on increasing access to maternal health services, improving birth preparedness, and investing in health financing. The initiative can also inform other countries that are seeking to encourage individual and family savings towards maternal health; increased birth preparedness and service utilization for maternal health care and involvement of male partners in maternal health were two areas of interest covered by this study. The research will contribute to the growing body of research connecting the importance of behavioral insights, empowerment, and human rights through health in a sustainable manner.

### Trial status

Trial enrollment is expected to start in June 2022 and end in December 2022. The trial is expected to be completed by June 2023.

## Supplementary Information

**Additional file 1. **SPIRIT 2013 Checklist.

## Data Availability

The data collection tools and datasets generated during the study are not publicly available due to confidentiality of participant data under financial transaction services under clinicPesa but will be made available by the corresponding author on reasonable request.

## Abbreviations

ANC: Antenatal care
CCT: Conditional cash transfer
MMR: Maternal mortality rate
MTN: Mobile telephone network
RA: Research assistant
RCT: Randomized controlled trial
SBA: Skilled birth attendance
SMS: Short message service
SPIRIT: Standard Protocol Items: Recommendations for Intervention Trials
SSA: Sub-Saharan Africa
UGX: Ugandan Shilling
USD: United States Dollar
